# Blind Separation of Skin Chromophores from Multispectral Dermatological Images

**DOI:** 10.3390/diagnostics14202288

**Published:** 2024-10-14

**Authors:** Mustapha Zokay, Hicham Saylani

**Affiliations:** Laboratory of Materials, Signals, Systems and Physical Modeling, Faculty of Sciences, Ibn Zohr University, Agadir BP 8106, Morocco; h.saylani@uiz.ac.ma

**Keywords:** Blind Source Separation, chromophores, melanin, oxyhemoglobin, deoxyhemoglobin, shading, multispectral dermatological images

## Abstract

**Background/Objectives**: Based on Blind Source Separation and the use of multispectral imaging, the new approach we propose in this paper aims to improve the estimation of the concentrations of the main skin chromophores (melanin, oxyhemoglobin and deoxyhemoglobin), while considering shading as a fully-fledged source. **Methods**: In this paper, we demonstrate that the use of the Infra-Red spectral band, in addition to the traditional RGB spectral bands of dermatological images, allows us to model the image provided by each spectral band as a mixture of the concentrations of the three chromophores in addition to that of the shading, which are estimated through four steps using Blind Source Separation. **Results**: We studied the performance of our new method on a database of real multispectral dermatological images of melanoma by proposing a new quantitative performances measurement criterion based on mutual information. We then validated these performances on a database of multispectral dermatological images that we simulated using our own new protocol. **Conclusions**: All the results obtained demonstrated the effectiveness of our new approach for estimating the concentrations of the skin chromophores from a multispectral dermatological image, compared to traditional approaches that consist of using only the RGB image by neglecting shading.

## 1. Introduction

Human skin is a complex tissue composed of three main chromophores, melanin, oxyhemoglobin and deoxyhemoglobin [1,2]. Information about the concentrations of these chromophores is crucial, as it plays a key role in the early detection of skin diseases and their monitoring throughout the period of therapeutic treatment [3,4,5,6]. To determine these concentrations, researchers are increasingly relying on RGB or multispectral imaging techniques (multispectral imaging involves acquiring different images of the same area of interest at various wavelengths) [3] rather than conventional techniques which are mostly invasive and generally more burdensome. Indeed, the traditional biopsy technique, involving the extraction of samples from the affected skin area for analysis in a laboratory, is an invasive procedure that is burdensome for both the patient and the practitioner, especially considering the possibility of needing to repeat it throughout the period of treatment.

Multispectral and hyperspectral imaging have become indispensable tools for detecting skin diseases through Computer-Aided Detection (CAD) models [7,8,9]. However, the major challenge encountered in the exploitation of multispectral dermatological images lies in establishing a link between the chromophores and the images acquired that is as rigorous as possible, and this has led to several ways of modeling these optical images and subsequently to multiple methods for their processing. We begin then in Section 1.1 by shedding light on the optical model most widely adopted by the scientific community for the treatment of dermatological images, and then, in the following Section 1.2, we review the existing methods situating them in relation to this model.

### 1.1. Physical Modeling of Dermatological Images

By adopting the optical model widely used by the scientific community known as the dichromatic model [10,11], the reflectance of the image can be represented as an additive mixture of diffuse reflection and specular reflection. Since the latter can be eliminated using polarizers [12] or image processing methods [13], only the diffuse reflection remains. Thus, according to Lambert–Beer’s law [14,15], the diffuse reflection, denoted as $I_{d}\left(\lambda ,u\right)$ for each wavelength λ, is described by the following equation:

(1) $$I_{d}\left(\lambda ,u\right)=G\left(\lambda \right)\cdot E_{d}\left(\lambda \right)\cdot w_{d}\left(u\right)\cdot e^{-2f(\lambda ,u)},$$

where $f\left(\lambda ,u\right)=\mu_{ox}\left(\lambda \right)\ell_{ox}\left(\lambda \right)C_{ox}\left(u\right)+\mu_{deox}\left(\lambda \right)\ell_{deox}\left(\lambda \right)C_{deox}\left(u\right)+\mu_{m}\left(\lambda \right)\ell_{m}\left(\lambda \right)C_{m}\left(u\right)$ and

- G(λ) represents a gain that characterizes the camera;
- $E_{d}\left(\lambda \right)$ is the intensity of the diffuse component of the light source;
- $w_{d}\left(u\right)$ models the variation of shading that may be present in the image;
- $\mu_{ox}\left(\lambda \right)$, $\mu_{deox}\left(\lambda \right)$ and $\mu_{m}\left(\lambda \right)$ are, respectively, dependent on the absorption coefficients of oxyhemoglobin, deoxyhemoglobin and melanin;
- $\ell_{ox}\left(\lambda \right)$, $\ell_{deox}\left(\lambda \right)$ and $\ell_{m}\left(\lambda \right)$ are, respectively, the optical paths for oxyhemoglobin, deoxyhemoglobin and melanin;
- $C_{ox}\left(\lambda \right)$, $C_{deox}\left(\lambda \right)$ and $C_{m}\left(\lambda \right)$ are, respectively, the concentrations of oxyhemoglobin, deoxyhemoglobin and melanin.

If we denote $I_{\lambda }\left(u\right)=-\log\left(I_{d}\left(\lambda ,u\right)\right)$, then Equation (1) yields

(2) $$\begin{array}{ccc} I_{\lambda }\left(u\right) & = & 2\mu_{ox}\left(\lambda \right).\ell_{ox}\left(\lambda \right).C_{ox}\left(u\right)+2\mu_{deox}\left(\lambda \right).\ell_{deox}\left(\lambda \right).C_{deox}\left(u\right)+2\mu_{m}\left(\lambda \right).\ell_{m}\left(\lambda \right).C_{m}\left(u\right)\\ & & -\log\left(G\left(\lambda \right)\right)-\log\left(E_{d}\left(\lambda \right)\right)-\log\left(w_{d}\left(u\right)\right) \end{array}$$

If we denote $\lambda_{i}$(i=1,2,3,⋯) as the wavelength corresponding to the spectral band of index *i* in the multispectral image and j∈{1,2,3} as the index associated to the concentrations of the three chromophores (oxyhemoglobin, deoxyhemoglobin and melanin), then we can rewrite Equation (2) in the following form:

(3) $$I_{\lambda_{i}}\left(u\right)=\sum_{j=1}^{j=3}m_{ij}.S_{j}\left(u\right)+p_{d}\left(u\right)+n_{i},\;i=1,2,3,\cdots$$

where

- $m_{ij}=2\mu_{j}\left(\lambda_{i}\right)\ell_{j}\left(\lambda_{i}\right)$,
- $S_{j}\left(u\right)=C_{j}\left(u\right)$,
- $p_{d}\left(u\right)=-\log\left(w_{d}\left(u\right)\right)$,
- $n_{i}=-\log\left(G\left(\lambda_{i}\right)\right)-\log\left(E_{d}\left(\lambda_{i}\right)\right)$.

Thus, for any spectral band with index *i*, the quantity $I_{\lambda_{i}}\left(u\right)$ is a mixture of the concentrations of the three chromophores $S_{j}\left(u\right)$, which we aim to estimate separately, along with the shading $p_{d}\left(u\right)$. It is therefore an inverse source separation problem, as we seek to separate the concentrations of the three chromophores $S_{j}\left(u\right)$, viewed as the sources of interest, knowing only their mixtures $I_{\lambda_{i}}\left(u\right)$. In the case where no a priori information about either the sources or the mixing coefficients is avalilable, it is referred to as Blind Source Separation (BSS). It is shown that, in this case, it is an ill-posed inverse problem, making it essential to introduce hypotheses on the sources and/or mixing coefficients. Depending on the nature of these hypotheses, three main families of BSS methods are distinguished. The BSS methods based on Independent Component Analysis (ICA), which exploit the hypothesis of independence between sources, those based on Sparse Component Analysis (SCA), which exploit the hypothesis of sparsity of the sources, and finally, the BSS methods based on Non-negative Matrix Factorization (NMF), which exploit the positivity of both sources and mixing coefficients (See [16,17] for more details). We then review, in the following subsequent section, the main existing methods that focus on estimating chromophore concentrations and that are mostly based on BSS.

### 1.2. Existing Methods

Based on the working hypotheses and the procedure followed to estimate the concentrations of different chromophores, we can classify the various existing methods into two main families. The first family includes methods that estimate chromophore concentrations by exploiting prior knowledge about the absorption spectra of each chromophore and the depths of light penetration into the skin [4,12,18,19,20,21]. Indeed, in [4,12,18,19,20], the authors first proposed to simplify the mixture model by eliminating shading and specular reflection through the use of white paper and polarizers to obtain a mixing model composed only of the three main chromophores. They then exploited the absorption spectra and light penetration depths into the skin to empirically estimate the mixing coefficients $m_{ij}$, which ultimately enable the estimation of the concentrations of the three chromophores. In [21], the authors adopted a very particular mixture model in that they completely neglected shading on the one hand and introduced an additional component alongside the three main chromophores on the other hand, representing the contribution of the absorption residue of any other molecule contained in the dermis and epidermis. However, they exploited prior knowledge exactly as in [4,12,18,19,20] to estimate the concentrations of the three chromophores after correcting the non-uniformity of brightness in dermatological images by transforming them into the CIEXYZ color space. We also note that their study focused on comparing two types of dermatological image acquisition cameras in terms of the quality of estimation of the concentrations of the different chromophores [21].

The second family groups the methods that treat the mixture model defined by Equation (3) as a three-source mixture model, considering oxyhemoglobin and deoxyhemoglobin as a single source called hemoglobin [22,23,24,25,26,27,28,29]. These methods focus on estimating the concentrations of melanin and hemoglobin from RGB images by using mainly BSS methods based on ICA or NMF. Depending on how shading is taken into account in the mixture model, we distinguish between two classes for these methods. Among the methods in the first class that neglected shading [22,23,24,27,29], those proposed by Tsumura et al. in [22,23,24] assumed the independence between melanin and hemoglobin, employing a BSS method based on ICA. In [29], Xinhua et al. proposed using the Shades of Grey method [30] as a pre-processing step to eliminate color interferences caused by illumination, and then used [23] to estimate the concentrations of these two chromophores. In [27], Gong et al. proposed two distinct BSS methods to estimate the concentrations of melanin and hemoglobin. Both methods are based on NMF using the popular algorithm for multiplicative updates of matrices solutions named Multiplicative Update [31]. Among the methods in the second class, which have taken shading into account in their mixture model, are those proposed in [25,26,28]. Based on ICA, the method proposed in [28] by Luisa et al. allows estimating the concentrations of both chromophores and shading, assuming they are all three independent. To improve the estimation of the concentrations of the two chromophores, Madooei et al. first proposed a technique exploiting the mean geometric to eliminate shading, and then they used ICA [26]. Liu et al. proposed in [25] to apply a bilateral filter to remove shading; then, they used the tables of extinction coefficients of the two chromophores and the depths of light penetration into the skin [32] to estimate their concentrations. However, it is noteworthy that all methods in this second family are limited to estimating hemoglobin in addition to melanin and therefore do not allow the estimation of oxyhemoglobin and deoxyhemoglobin separately.

In this article, we propose a novel method for estimating the concentrations of the three main chromophores (i.e., melanin, oxyhemoglobin and deoxyhemoglobin) in the skin by adopting a mixture model that is much more rigorous than existing models [33,34] and a separation method that is entirely blind. Indeed, assuming that the model given by Equation (3) is not perfectly respected regarding the contribution of shading, due to possible modeling errors, we consider shading as a fully fledged source in the same way as the concentrations of the three chromophores. As this hypothesis gives rise to mixtures of four sources, it is necessary to have multispectral dermatological images with at least four spectral bands. On the other hand, the method we propose is entirely blind compared to existing methods that rely on prior knowledge related to the mixing coefficients $m_{ij}$, which can vary depending on the light penetration depth into the skin (for example, epidermal thickness, which is the first layer of the skin where melanin is located, ranges from 76.9±26.2 to 267.4±120.6 μm, while the dermis thickness, the second layer of the skin where oxyhemoglobin and deoxyhemoglobin are found, ranges from 2115±946.4 to 4717.1±1902.5 μm [35]. Therefore, the penetration depth of light in the skin can vary from person to person). The new method we propose in this article is an extension of our recent work published in [36]. This extension covers both the “method” aspect and the “validation tests” aspect. Indeed, in terms of method, we propose a new processing step aimed at improving the final versions of the estimated chromophore concentrations. This processing consists of applying NMF to the matrix formed by these concentrations, thus remedying the problem of negative pixels encountered in the concentrations estimated by the basic version of our method proposed in [36]. Furthermore, we expand our validation tests on the one hand by increasing the number of real images processed, and on the other hand by also processing artificial multispectral images generated using a new simulation protocol that we developed. Indeed, validating our new method on artificial images allows us to assess the validity of our mixture model and working hypotheses. Finally, we also propose a new performance measurement criterion that is much more rigorous than the one proposed in [36] in the case of real dermatological images. The remainder of this article is structured as follows. Section 2 describes in detail our new method for estimating the concentrations of the three chromophores and shading. Section 3 presents the results of the tests carried out, followed by a discussion and a concluding section dedicated to summarizing our work and outlining future directions.

## 2. Proposed Method

Our method is principally distinguished by a first key idea which consists of considering shading as a fully fledged source, which we denote as $S_{4}\left(u\right)$, in the same way as the three sources of interest, i.e., the three chromophores. This idea is founded in the fact that in practice, due to modeling errors (which are closely related to image acquisition conditions), the contribution of shading cannot be exactly the same in all spectral bands, as ideally formulated in Equation (3). Our idea was in fact supported by experimental results relative to the estimation of principal components contained in biomedical images processed in [37]. These results demonstrate that the contribution of shading varies as a function of spectral bands and does not follow a strict uniformity, but rather exhibits slight variations along the reflectance ranges from 500 to 800 nm. Thus, by substituting the component $p_{d}\left(u\right)$ with $m_{i4}S_{4}\left(u\right)$ in Equation (3), where $m_{i4}$ is a real coefficient, we obtain

(4) $$I_{\lambda_{i}}\left(u\right)=\sum_{j=1}^{j=3}m_{ij}\cdot S_{j}\left(u\right)+m_{i4}\cdot S_{4}\left(u\right)+n_{i},\;i=1,2,3,\cdots$$

On the other hand, as in [23], the quantities $n_{i}$ can be estimated and subtracted from the mixtures. Indeed, assuming that there is at least one pixel where the concentrations of the three chromophores and shading are all zero, these quantities are given by the following relation:

(5) $$n_{i}=min\left(I_{\lambda_{i}}\left(u\right)\right).$$

So, by denoting $X_{i}\left(u\right)=I_{\lambda_{i}}\left(u\right)-n_{i}$, we obtain

(6) $$X_{i}\left(u\right)=\sum_{j=1}^{j=4}m_{ij}\cdot S_{j}\left(u\right),\;i=1,2,3,\cdots$$

**Figure 1:**
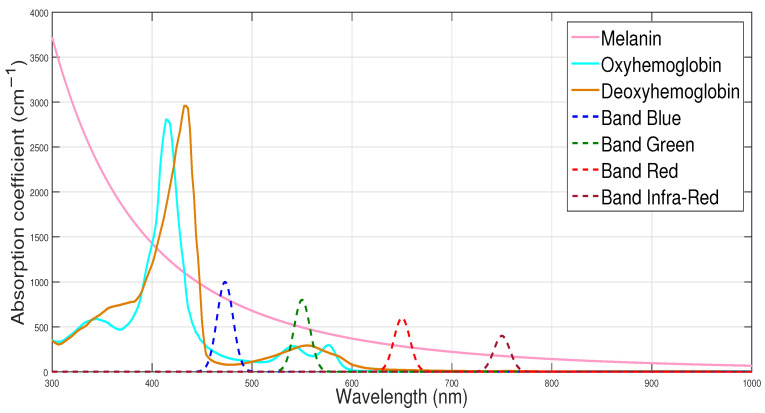
Chromophore absorption coefficient [25,39] and the 4 spectral bands of multispectral imaging.

The second key idea of our method is based on the exploitation of the *Infra-Red* band as a fourth spectral band in addition to the classic *Red*, *Green* and *Blue* bands. On one hand, as we now have four sources to identify, we need at least four mixtures of these sources provided, respectively, by the four spectral bands (in this case, we refer to so-called determined mixtures, meaning there are as many mixtures as sources). On the other hand, our choice of the *Infra-Red* band as the fourth band, which was not made randomly, is based on the exploitation of properties related to the behavior of the absorption coefficients of the chromophores when the wavelength increases. In fact, we relied on the observations of Zhao et al. [25,38], who noted, according to the data illustrated in Figure 1, that the absorption coefficients $m_{ij}$ of oxyhemoglobin and deoxyhemoglobin are negligible compared to melanin for wavelengths greater than 620 nm, meaning ideally that [25,38]

(7) $$\left\{\begin{array}{c} m_{31}=m_{32}=0\\ m_{41}=m_{42}=0 \end{array}\right.$$

So, Equation (6) for the four selected spectral bands offers us

(8) $$\left\{\begin{array}{c} X_{1}\left(u\right)=m_{11}\cdot S_{1}\left(u\right)+m_{12}\cdot S_{2}\left(u\right)+m_{13}\cdot S_{3}\left(u\right)+m_{14}\cdot S_{4}\left(u\right)\\ X_{2}\left(u\right)=m_{21}\cdot S_{1}\left(u\right)+m_{22}\cdot S_{2}\left(u\right)+m_{23}\cdot S_{3}\left(u\right)+m_{24}\cdot S_{4}\left(u\right)\\ X_{3}\left(u\right)=m_{33}\cdot S_{3}\left(u\right)+m_{34}\cdot S_{4}\left(u\right)\\ X_{4}\left(u\right)=m_{43}\cdot S_{3}\left(u\right)+m_{44}\cdot S_{4}\left(u\right) \end{array}\right.$$

Taking these elements into account, the method we propose, which aims to estimate the concentrations of the three chromophores separately, proceeds in four steps. During the first step, we focus on mixtures $X_{3}\left(u\right)$ and $X_{4}\left(u\right)$ to estimate the concentrations of melanin and shading. Subsequently, in the second step, we propose a procedure to properly subtract their contributions from the first two mixtures $X_{1}\left(u\right)$ and $X_{2}\left(u\right)$, allowing us to focus on the concentrations of oxyhemoglobin and deoxyhemoglobin in the third step. Based on NMF, the fourth step aims to improve the estimations of the concentrations of the three chromophores provided at the end of the first three steps of our method. These four steps are the subject of the following four subsequent sections, respectively.

### 2.1. Estimation of Sources $S_{3}\left(u\right)$ and $S_{4}\left(u\right)$

During this first step, we only exploit the mixtures $X_{3}\left(u\right)$ and $X_{4}\left(u\right)$ provided by the *Red* and *Infra-Red* spectral bands, which can be expressed as

(9) $$\left\{\begin{array}{c} X_{3}\left(u\right)=m_{33}\cdot S_{3}\left(u\right)+m_{34}\cdot S_{4}\left(u\right)\\ X_{4}\left(u\right)=m_{43}\cdot S_{3}\left(u\right)+m_{44}\cdot S_{4}\left(u\right) \end{array}\right.$$

This system of Equation (9) can be formulated in the matrix form as follows:

(10) $$X_{34}\left(u\right)=M_{34}\cdot S_{34}\left(u\right),$$

where $X_{34}\left(u\right)={\left[X_{3}\left(u\right),X_{4}\left(u\right)\right]}^{T}$, $S_{34}\left(u\right)={\left[S_{3}\left(u\right),S_{4}\left(u\right)\right]}^{T}$ and $M_{34}=\left(\begin{array}{cc} m_{33} & m_{34}\\ m_{43} & m_{44} \end{array}\right)$.

The aim is therefore to estimate the matrix $M_{34}^{-1}$, called the unmixing matrix, in order to recover the source matrix $S_{34}\left(u\right)$ and subsequently the two sources $S_{3}\left(u\right)$ and $S_{4}\left(u\right)$. Knowing that melanin depends only on the diffusion of matter by the melanocyte cells while shading depends only on the geometry of the skin, which can be described by the dot product between the surface normal vector of the skin and the lighting direction vector. This geometry influences the concentration of light at the surface, leading us to deduce that the two corresponding sources $S_{3}\left(u\right)$ and $S_{4}\left(u\right)$ are independent. Thus, to separate them, we can effectively use one of the BSS methods based on ICA. As these two sources are auto-correlated, an ICA method exploiting second-order statistics is sufficient. In this regard, we opted here for the popular Algorithm for Multiple Unknown Signals Extraction (AMUSE) [40], known for its simplicity and efficiency, as long as the following working hypotheses are verified:

**Hypothesis** **1.** *The sources $S_{3}\left(u\right)$ and $S_{4}\left(u\right)$ are mutually uncorrelated, i.e.,*

(11) $$\forall \;v,\;E\left[S_{3}\left(u\right)\cdot S_{4}\left(u-v\right)\right]=E\left[S_{3}\left(u\right)\right]\cdot E\left[S_{4}\left(u-v\right)\right]$$

**Hypothesis** **2.** *The sources $S_{3}\left(u\right)$ and $S_{4}\left(u\right)$ satisfy the following identifiability condition:*

(12) $$\exists \;\;v\neq 0\;\;/\;\frac{E[S_{3}\left(u\right)\cdot S_{3}\left(u-v\right)]}{E[S_{3}^{2}\left(u\right)]}\neq \frac{E[S_{4}\left(u\right)\cdot S_{4}\left(u-v\right)]}{E[S_{4}^{2}\left(u\right)]}$$

It is then shown that if these working hypotheses are satisfied, the AMUSE method [40] ultimately allows estimating the unmixing matrix $M_{34}^{-1}$ up to a permutation matrix and a diagonal matrix, denoted respectively, as P and D. Denoted as U, the estimated unmixing matrix by the AMUSE method is then expressed as [40]

(13) $$U={\mathrm{PDM}}_{34}^{-1}.$$

After estimating the unmixing matrix U, in addition to the centered sources denoted as ${\tilde{S}}_{j}\left(u\right)$ usually estimated by the AMUSE method, we propose in this step to also estimate the non-centered sources $S_{j}\left(u\right)$, which are needed in the second step. Indeed, according to Equations (10) and (13), the product $U\cdot X_{34}\left(u\right)$, denoted as Y(u), is expressed as

(14) $$\begin{array}{ccc} Y(u) & = & \left({\mathrm{PDM}}_{34}^{-1}\right)\left(M_{34}S_{34}\left(u\right)\right) \end{array}$$

(15) $$\begin{array}{ccc} & = & \mathrm{PD}\cdot S_{34}\left(u\right) \end{array}$$

Thus, we obtain an estimation of the source matrix $S_{34}\left(u\right)$ with the same indeterminacies as the unmixing matrix $M_{34}^{-1}$. On the other hand, by writing $Y\left(u\right)=[Y_{3}\left(u\right),Y_{4}\left(u\right)]$ and knowing that in our case the permutation matrix P can be reduced to the identity matrix, as we can easily differentiate the distribution of shading (due to its nature) from that of melanin, we obtain Equation (16). Indeed, in BSS, the permutation matrix P is introduced solely to specify that the order of the estimated sources (i.e., $Y_{3}\left(u\right)$ then $Y_{4}\left(u\right)$) is not necessarily the same as that in their matrix (i.e., $S_{3}\left(u\right)$ for melanin and $S_{4}\left(u\right)$ for shading), which was initially arbitrarily fixed, and this does not pose any problems.

(16) $$Y_{j}\left(u\right)=d_{j}\cdot S_{j}\left(u\right),\;j=3,4$$

where the $d_{j}$ are the two coefficients contained in the diagonal of the matrix D. Furthermore, according to this Equation (16), the centered versions of these signals $Y_{j}\left(u\right)$, denoted as ${\tilde{Y}}_{j}\left(u\right)$, are expressed as

(17) $${\tilde{Y}}_{j}\left(u\right)=d_{j}\cdot {\tilde{S}}_{j}\left(u\right),\;j=3,4$$

### 2.2. Removing Sources $S_{3}\left(u\right)$ and $S_{4}\left(u\right)$ from Mixtures

In this step, our goal is to remove from the mixtures $X_{1}\left(u\right)$ and $X_{2}\left(u\right)$ the contributions of the sources we just estimated, $S_{3}\left(u\right)$ and $S_{4}\left(u\right)$. To achieve this, we rely on the following independence hypothesis:

**Hypothesis** **3.** *The sources $S_{i}\left(u\right)$ (i=1,2) and $S_{j}\left(u\right)$ (j=3,4) are mutually uncorrelated instantaneously, i.e.,*

(18) $$E\left[{\tilde{S}}_{i}\left(u\right)\cdot {\tilde{S}}_{j}\left(u\right)\right]=E\left[{\tilde{S}}_{i}\left(u\right)\right]\cdot E\left[{\tilde{S}}_{j}\left(u\right)\right]=0,\;\forall \left(i,j\right)\in \left\{1,2\right\}\times \left\{3,4\right\}$$

By using Equations (17) and (18), we can write

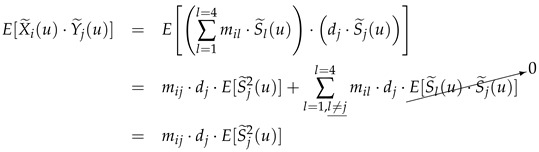
(19)

On the other hand, by referring to Equation (17), we can write

(20) $$E\left[{\tilde{Y}}_{j}^{2}\left(u\right)\right]=d_{j}^{2}\cdot E\left[{\tilde{S}}_{j}^{2}\left(u\right)\right].$$

Thus, we have

(21) $$\frac{E[{\tilde{X}}_{i}\left(u\right)\cdot {\tilde{Y}}_{j}\left(u\right)]}{E[{\tilde{Y}}_{j}^{2}\left(u\right)]}=\frac{m_{ij}\cdot d_{j}}{d_{j}^{2}}=\frac{m_{ij}}{d_{j}}.$$

From the basic mixtures $X_{1}\left(u\right)$ and $X_{2}\left(u\right)$ expressed in System (8) and using Equations (16) and (21), we can create two new mixtures that contain only the sources $S_{1}\left(u\right)$ and $S_{2}\left(u\right)$. Denoted as $X_{i}^{\prime }\left(u\right)$ (i=1,2), these new mixtures are obtained as follows:

(22) $$\begin{array}{ccc} X_{i}^{\prime }\left(u\right) & = & X_{i}\left(u\right)-\frac{E[{\tilde{X}}_{i}\left(u\right)\cdot {\tilde{Y}}_{3}\left(u\right)]}{E[{\tilde{Y}}_{3}^{2}\left(u\right)]}\cdot Y_{3}\left(u\right)-\frac{E[{\tilde{X}}_{i}\left(u\right)\cdot {\tilde{Y}}_{4}\left(u\right)]}{E[{\tilde{Y}}_{4}^{2}\left(u\right)]}\cdot Y_{4}\left(u\right)\\ & = & X_{i}\left(u\right)-\frac{m_{i3}}{d_{3}}\cdot \left(d_{3}.\cdot S_{3}\left(u\right)\right)-\frac{m_{i4}}{d_{4}}\cdot \left(d_{4}\cdot S_{4}\left(u\right)\right)\\ & = & m_{i1}\cdot S_{1}\left(u\right)+m_{i2}\cdot S_{2}\left(u\right) \end{array}$$

Hence,

(23) $$\left\{\begin{array}{c} X_{1}^{\prime }\left(u\right)=m_{11}\cdot S_{1}\left(u\right)+m_{12}\cdot S_{2}\left(u\right)\\ X_{2}^{\prime }\left(u\right)=m_{21}\cdot S_{1}\left(u\right)+m_{22}\cdot S_{2}\left(u\right) \end{array}\right.$$

### 2.3. Estimation of Sources $S_{1}\left(u\right)$ and $S_{2}\left(u\right)$

The objective of this step is to estimate the remaining sources $S_{1}\left(u\right)$ and $S_{2}\left(u\right)$, representing the concentrations of oxyhemoglobin and deoxyhemoglobin, respectively, from the new mixtures $X_{1}^{\prime }\left(u\right)$ and $X_{2}^{\prime }\left(u\right)$ expressed in the system of Equation (23). However, as these two sources are statistically dependent, since they both come from the same source, i.e., blood, no BSS method based on ICA can be used to separate them. This is why in this article we propose to explore another alternative, which is to exploit the sparsity of these two sources to separate them, unlike all the existing methods that focus on estimating the concentrations of the different chromophores using BSS. Here, then, is our working hypothesis relating to the exploitation of the sparsity of the sources $S_{1}\left(u\right)$ and $S_{2}\left(u\right)$.

**Hypothesis** **4.** *For each source $S_{j}\left(u\right)$(j=1,2), there exists at least one spatial zone, denoted $U_{j},$ where it is present in both mixtures $X_{1}^{\prime }\left(u\right)$ and $X_{2}^{\prime }\left(u\right)$, i.e.,*

(24) $$\begin{array}{ccc} \exists \;\;U_{1} & / & \forall \;u\in U_{1},\;X_{1}^{\prime }\left(u\right)=m_{11}S_{1}\left(u\right)\;\mathrm{and}\;X_{2}^{\prime }\left(u\right)=m_{21}S_{1}\left(u\right) \end{array}$$

(25) $$\begin{array}{ccc} \exists \;\;U_{2} & / & \forall \;u\in U_{2},\;X_{1}^{\prime }\left(u\right)=m_{12}S_{2}\left(u\right)\;\mathrm{and}\;X_{2}^{\prime }\left(u\right)=m_{22}S_{2}\left(u\right) \end{array}$$

Note that there are several BSS methods that exploit this hypothesis of source sparsity [41,42]. In this study, we opted for the *TEMPROM* method (here, in this context, ‘Temp’ and ‘Rom’ represent spatial ratios rather than temporal ones. These terms do not refer to temporal aspects but rather to spatial relations between sources) [41], known for its simplicity in terms of implementation and effectiveness. It is based on exploiting the ratio between mixtures $X_{2}^{\prime }\left(u\right)$ and $X_{1}^{\prime }\left(u\right)$ to identify zones $U_{j}$ qualified as mono-source zones. Indeed, the calculation of this ratio in these zones $U_{j}$, denoted as $r_{j}$, yields

(26) $$\begin{array}{c} \forall \;u\in U_{1},\;\;\;\frac{X_{2}^{\prime }\left(u\right)}{X_{1}^{\prime }\left(u\right)}=\frac{m_{21}S_{1}\left(u\right)}{m_{11}S_{1}\left(u\right)}=\frac{m_{21}}{m_{11}}=r_{1} \end{array}$$

(27) $$\begin{array}{c} \forall \;u\in U_{2},\;\;\;\frac{X_{2}^{\prime }\left(u\right)}{X_{1}^{\prime }\left(u\right)}=\frac{m_{22}S_{2}\left(u\right)}{m_{12}S_{2}\left(u\right)}=\frac{m_{22}}{m_{12}}=r_{2} \end{array}$$

By exploiting Equations (23), (26) and (27), we can write

(28) $$\left\{\begin{array}{c} r_{2}\cdot X_{1}^{\prime }\left(u\right)-X_{2}^{\prime }\left(u\right)=\left(r_{2}\cdot m_{11}-m_{21}\right)\cdot S_{1}\left(u\right)=d_{1}\cdot S_{1}\left(u\right)\\ r_{1}\cdot X_{1}^{\prime }\left(u\right)-X_{2}^{\prime }\left(u\right)=\left(r_{1}\cdot m_{12}-m_{22}\right)\cdot S_{2}\left(u\right)=d_{2}\cdot S_{2}\left(u\right) \end{array}\right.$$

where $d_{1}=r_{2}\cdot m_{11}-m_{21}$ and $d_{2}=r_{1}\cdot m_{12}-m_{22}$. Thus, we obtain each of the two sources $S_{j}\left(u\right),\left(j=1,2\right)$ up to the coefficient $d_{j}$.

However, it remains necessary to discuss how the identification of the different mono-source zones $U_{1}$ and $U_{2}$ and the estimation of the ratios $r_{1}$ and $r_{2}$ are performed, and then the differentiation between them. To address this, we propose an enhanced version of the basic TEMPROM method [41]. Initially, as in [41], we use a criterion based on the calculation of the variance of the ratio $\frac{X_{2}^{\prime }\left(u\right)}{X_{1}^{\prime }\left(u\right)}$. Indeed, it is evident that the variance of this ratio is zero over any mono-source zone according to Equations (26) and (27). In this regard, we begin by subdividing the mixtures into several segments of equal length *L* and calculating the variance of the ratio for each of these segments. Any segment with negligible variance (having a value below a user-defined threshold in practice) is retained as a mono-source zone, resulting in a finite number denoted as *K* of mono-source zones. The set of these *K* mono-source zones, denoted as $U^{\left(k\right)}\left(k=1,2,\cdots ,K\right),$ is thus constituted of two parts, one part of zones is of type $U_{1}$ and another part of zones of type $U_{2}$. On each of these zones $U^{\left(k\right)}$, we then estimate the mean of the ratio $\frac{X_{2}^{\prime }\left(u\right)}{X_{1}^{\prime }\left(u\right)}$, denoted as R(k), as follows:

(29) $$R\left(k\right)=E\left[\frac{X_{2}^{\prime }\left(u\right)}{X_{1}^{\prime }\left(u\right)}\right],\;\forall \;u\in U^{\left(k\right)},\;k=1,2,\cdots ,K.$$

These *K* mono-source zones are then ranked based on their variances in ascending order. Obviously, the first ranked zone, referred to as the best mono-source zone, provides us with an estimation of $r_{1}$ or $r_{2}$, i.e.,

(30) $$R\left(1\right)={\hat{r}}_{1}\;\mathrm{or}\;R\left(1\right)={\hat{r}}_{2}.$$

Since the second mono-source zone $U^{\left(2\right)}$ could very well be of the same type as the first one, and thus provide the estimation of the same ratio $r_{i}$, i.e., R(2)=R(1), the authors of the basic method [41] have proposed exploring the set of zones $U^{\left(k\right)}$ until finding a ratio R(k) such that |R(k)−R(1)|⩾e, where *e* is a threshold which is fixed heuristically, in which case they associate R(k) with the estimation of the second ratio $r_{j}$ as follows:

(31) $$R\left(k\right)={\hat{r}}_{2}\;\mathrm{if}\;R\left(1\right)={\hat{r}}_{1}\;\mathrm{or}\;R\left(k\right)={\hat{r}}_{1}\;\mathrm{if}\;R\left(1\right)={\hat{r}}_{2}.$$

However, their heuristic choice for the threshold *e* may potentially degrade the method, as its true value, which is equal to $|r_{2}-r_{1}|$, is typically unknown and varies from one dermatological image to another. The alternative we propose in this article to address this issue is to introduce a variable threshold *e* that adjusts automatically according to the processed dermatological image. Indeed, we chose for this threshold *e* the value $\frac{R_{min}+R_{max}}{2}$, corresponding to the midpoint of the interval $\left[R_{min},R_{max}\right]$. This method provides a greater likelihood of obtaining an estimation of the second ratio $r_{j}$ that is dissimilar to that of the first ratio $r_{i(i\neq j)}$, where $R_{min}=min\left\{R(k)\right\}$ and $R_{max}=max\left\{R(k)\right\}$ for k∈[1,K].

Finally, if we take up Equation (28), which provides us with estimates of the concentrations of oxyhemoglobin ($S_{1}\left(u\right)$) and deoxyhemoglobin ($S_{2}\left(u\right)$), we see that we need to propose a method to determine which of the two estimated ratios R(1) and R(k) is ${\hat{r}}_{1}$ (which allows us to find the source $S_{2}\left(u\right)$) and which one is ${\hat{r}}_{2}$ (which allows us to find the source $S_{1}\left(u\right)$). To address this potential permutation problem of the estimated sources, we propose to use the absorption spectra of these two chromophores represented in Figure 1, since the absorption spectra of oxyhemoglobin informs us about the ratio ${\hat{r}}_{1}⋍\frac{m21}{m11}$, while that of deoxyhemoglobin provides information about the ratio ${\hat{r}}_{2}⋍\frac{m22}{m12}$. Based on these spectra and considering that the mixtures we exploited ($X_{1}^{\prime }\left(u\right)$ and $X_{2}^{\prime }\left(u\right)$) to estimate these two sources correspond, respectively, to the blue and green spectral bands, we deduce easily that ${\hat{r}}_{2}>{\hat{r}}_{1}$, allowing us to write Equation (32). Indeed, we recall, on one hand, that the mixture coefficients $m_{ij}$ are expressed as $m_{ij}=2\mu_{j}\left(\lambda_{i}\right)\ell_{j}\left(\lambda_{i}\right)$, where $\mu_{j}\left(\lambda_{i}\right)$ is the absorption coefficient of the chromophore $S_{j}\left(u\right)$ at the wavelength $\lambda_{i}$ (represented by one of the curves in Figure 1), and $\ell_{j}\left(\lambda_{i}\right)$ represents the optical path of this chromophore. On the other hand, we note that the optical paths of light passing through the two chromophores $S_{1}\left(u\right)$ and $S_{2}\left(u\right)$ (i.e., oxyhemoglobin and deoxyhemoglobin) are nearly the same (as they are both located at the same layer of the skin). Thus, based on Figure 1, focusing on the blue bands (i.e., $\lambda_{1}\in \left[453,473\right]$ nm) and green bands (i.e., $\lambda_{2}\in \left[550,570\right]$ nm), we easily deduce that $\frac{\mu_{2}\left(\lambda_{2}\right)}{\mu_{2}\left(\lambda_{1}\right)}>\frac{\mu_{1}\left(\lambda_{2}\right)}{\mu_{1}\left(\lambda_{1}\right)}$, and consequently, $\frac{m_{22}}{m_{12}}>\frac{m_{21}}{m_{11}}$.

(32) $${\hat{r}}_{2}=max\left\{R(1),R(k)\right\}\;\mathrm{and}\;{\hat{r}}_{1}=min\left\{R(1),R(k)\right\}.$$

So, if we define $Y_{1}\left(u\right)={\hat{r}}_{2}\cdot X_{1}^{\prime }\left(u\right)-X_{2}^{\prime }\left(u\right)$ and $Y_{2}\left(u\right)={\hat{r}}_{1}\cdot X_{1}^{\prime }\left(u\right)-X_{2}^{\prime }\left(u\right)$; then, according to Equation (28), we have:

(33) $$Y_{j}\left(u\right)⋍d_{j}\cdot S_{j}\left(u\right),\;\;\;j=1,2.$$

This equation, in addition to Equation (16), allows us to finally conclude that each of the four sources $S_{j}\left(u\right)$ is estimated up to the coefficient $d_{j}$(j=1,2,3,4). Thus, if we denote Δ as the diagonal matrix such that $\Delta \left(j,j\right)=d_{j}$, then we can express this result in matrix form as follows:

(34) Y=Δ·S+E,

where $Y={\left[Y_{1}\left(u\right),Y_{2}\left(u\right),Y_{3}\left(u\right),Y_{4}\left(u\right)\right]}^{T}$, $S={\left[S_{1}\left(u\right),S_{2}\left(u\right),S_{3}\left(u\right),S_{4}\left(u\right)\right]}^{T}$ and E is a matrix of the same size as the matrix S that models the deviations due to cumulative estimation errors during the first three steps of our method. These errors, which may manifest as the presence of negative samples in the estimated concentrations of chromophores, are not tolerable, as the concentration is always non-negative (i.e., $S_{j}\left(u\right)⩾0$, ∀u). Therefore, in the next step, we propose to enhance the estimation obtained for each of the four sources by using Non-negative Matrix Factorization (NMF).

### 2.4. Improvement of Source Estimations Using NMF

The goal of this step is to improve the estimation of each source, including the concentrations of the chromophores of interest, by minimizing the errors incurred in the three previous steps. These expected errors are primarily due to the fact that the working hypotheses made about the sources (in terms of independence and sparsity) during these steps are not perfectly validated. For this reason, in this article, we opt for an additional step of *BSS*, which exploits the non-negativity of the sources $S_{j}\left(u\right)$ and the mixing coefficients $m_{ij}$. This involves a BSS step based on *Non-negative Matrix Factorization* (*NMF*) of the matrix formed by the initial mixtures $X_{i}\left(u\right)$, denoted as X and which, according to the system of Equation (8), is expressed as

(35) $$\begin{array}{ccc} X & = & M\cdot S, \end{array}$$

where $\;X={\left[X_{1}\left(u\right),X_{2}\left(u\right),X_{3}\left(u\right),X_{4}\left(u\right)\right]}^{T}$, $S={\left[S_{1}\left(u\right),S_{2}\left(u\right),S_{3}\left(u\right),S_{4}\left(u\right)\right]}^{T}$ and $M={\left(m_{ij}\right)}_{1⩽i,j⩽4}$.

We recall that BSS methods based on NMF seek to decompose the matrix X into two non-negative matrices N and H that best approximate the matrices M and S, respectively, i.e.,

(36) X=M·S=N·H,

with

(37) N⋍MandH⋍S.

As for the determination of matrices N and H, it is achieved through the minimization of a criterion based on measuring the deviation between the matrix X and the matrix product NH. Given that there are several criteria to measure this deviation [43], we opted here for the most commonly used criterion by the *BSS* methods based on the *NMF*, namely the Euclidean Distance between matrices X and NH, denoted as $D_{euc}\left(X|\mathrm{NH}\right)$ and defined by

(38) $$D_{euc}\left(X|\mathrm{NH}\right)=\frac{1}{2}||X-{NH||}^{2}.$$

There are various algorithms for minimizing this criterion [31,44]. We opted here for the *Alternating Least Squares* (*ALS*) algorithm [45], which is known both for its simplicity in terms of implementation and for its good performance, particularly in terms of speed [46]. By using this algorithm, the two solution matrices H and N are updated as follows [45]:

(39) $$\begin{array}{ccc} N & ⟵ & XH^{T}{\left(HH^{T}\right)}^{-1} \end{array}$$

(40) $$\begin{array}{ccc} H & ⟵ & {\left(N^{T}N\right)}^{-1}N^{T}X \end{array}$$

We point out, however, that the *NMF* is known for the problem of non-uniqueness of the factorization provided by all its algorithms. One of the solutions to this problem that has been proposed in the literature is to replace the random initialization of the solution matrices N and H by a particular initialization generally chosen by exploiting a priori knowledge of at least one of the two matrices being sought. It is shown in [47] that initializing at least one of the two solution matrices (N or H) with a version close to the corresponding sought matrix (M or S) generally makes it possible to reduce the set of solutions (infinite number) to a subset restricted to so-called permissible solutions, ideally expressed as follows:

(41) $$N=M{\left(\Pi \Lambda \right)}^{-1}\;\mathrm{and}\;H=\left(\Pi \Lambda \right)S,$$

where Π and Λ are, respectively, a permutation matrix and a diagonal matrix. In this article, to solve the non-uniqueness problem of the factorization, we propose to exploit the knowledge of an approximate version of the matrix S, specifically the one estimated at the end of the third step of our method. In other words, we propose initializing the matrix H with the matrix Y given by Equation (34). The update of the two matrices N and H through Equations (39) and (40) is then performed until the convergence of the *ALS* algorithm. In practice, this convergence is considered to be achieved when the difference between the matrix X and the product NH is less than a predetermined threshold ϵ.

Furthermore, by adopting the same approach as in Section 2.3 to solve the possible permutation problem of the estimated sources, we can rearrange the elements of the matrix H, provided in Equation (41), so that they correspond to the sources $S_{j}\left(u\right),\left(j=1,2,3,4\right)$ in the same order. Thus, we can omit the permutation matrix Π appearing in these equations and keep only the diagonal matrix Λ which reflects the fact that each source $S_{j}\left(u\right)$ is estimated up to a scaling factor equal to Λ(j,j). Therefore, Equation (41) offers us

(42) $$n_{ij}=m_{ij}\cdot \Lambda {\left(j,j\right)}^{-1}\;\mathrm{and}\;H_{j}\left(u\right)=\Lambda \left(j,j\right)\cdot S_{j}\left(u\right).$$

On the other hand, in order to overcome this scaling factor problem, which can vary from one source to another and from one dermatological image to another, we are rather interested in the contribution of each source $S_{j}\left(u\right)$ at each spectral band indexed by “*i*”, which is equal to $n_{ij}\cdot H_{j}\left(u\right)$, and then in the sum of these contributions, which is written according to Equation (42):

(43) $$\sum_{i=1}^{i=4}n_{ij}H_{j}\left(u\right)=\sum_{i=1}^{i=4}m_{ij}S_{j}\left(u\right).$$

**Figure 2:**
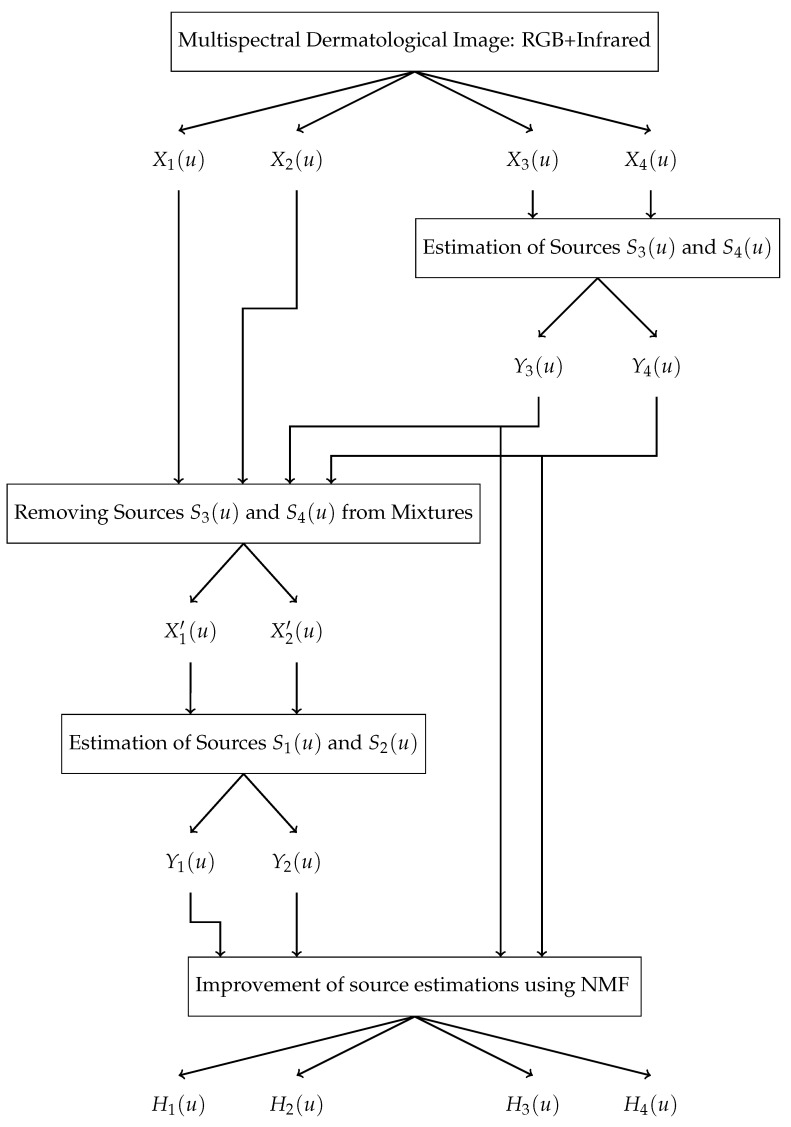
Flowchart illustrating the main steps of our method *BCSnmf-Irgb*.

Henceforth, we refer to the contribution of a source $S_{j}\left(u\right)$ to designate the summation $\sum_{i=1}^{i=4}m_{ij}S_{j}\left(u\right)$, and this by abuse of language. Furthermore, as our new method to Blind Chromophore Separation (BCS) of the skin in the presence of shading, by exploiting the Infrared band in addition to the three RGB spectral bands, differs from the one proposed in [36] by the important step of NMF, we call the resulting method “*BCSnmf-Irgb*”. However, we call the method resulting from our method at the third step (which we can qualify as a partial method) “*BCS-Irgb*”. Finally, the main aspects of our method, *BCSnmf-Irgb*, are summarized in the flowchart presented in Figure 2.

## 3. Results

In this section, we study the performance of our new method for estimating the concentrations of the main skin chromophores from multispectral dermatological images containing shading. For this, we first use a database of real multispectral dermatological images and then a database of artificial multispectral dermatological images that we developed in order to validate our results in terms of working hypotheses. On the other hand, as mentioned in Section 1.2, in absolute terms, none of the existing methods in the literature can be used for comparative purposes. We point out that all the Signal Processing (and/or BSS) methods focused on estimating skin chromophore concentrations are limited to estimating melanin and hemoglobin, which is a mixture of oxyhemoglobin and deoxyhemoglobin (see Section 1.2). Consequently, these methods do not allow for a comprehensive comparison. However, we can still study the performance of our method at the third step only through our method, *BCS-Irgb* [36], in order to highlight the interest of the fourth step of NMF of our global method through our new method *BCSnmf-Irgb*. Finally, in order to highlight the interest of considering shading as a fully fledged source, we are also interested in estimating the concentrations of the three chromophores from the RGB dermatological image, i.e., by neglecting the presence of shading. For this, we exploit only the second and third steps of our method, which can be seen as a subversion of our global method and which we call method “*BCS-rgb*” for the rest of this article.

For performance evaluation, in addition to the one based on the visual analysis of the representations of estimated concentrations, which is qualitative and therefore subjective, we propose a new criterion based on measuring the independence between melanin concentration and the concentrations of two other chromophores (oxyhemoglobin and deoxyhemoglobin) using the mutual information. Indeed, unlike the criterion used in [36], which exploits the four-order cross moments, our new criterion based on mutual information is invariant with regard to the scaling factors which are always present in the estimated concentrations of chromophores and typically vary from one dermatological image to another. It is important to note that the criterion for measuring the independence by exploiting the four-order cross moments, as defined in [36], is sensitive to scale factors and does not allow for a rigorous comparison between different chromophores in terms of independence. We recall that the mutual information between two random vectors $V_{i}$ and $V_{j}$ of the same length *N*, denoted as $I(V_{i};V_{j})$, is defined as [48]

(44) $$I\left(V_{i};V_{j}\right)=\sum_{m=1}^{N}\sum_{n=1}^{N}p\left(V_{i}^{\left(m\right)},V_{j}^{\left(n\right)}\right)\;\log_{2}\left(\frac{p(V_{i}^{\left(m\right)},V_{j}^{\left(n\right)})}{p\left(V_{i}^{\left(m\right)}\right)p\left(V_{j}^{\left(n\right)}\right)}\right),$$

where $p(V_{i}^{\left(m\right)},V_{j}^{\left(n\right)})$, $p(V_{i}^{\left(m\right)})$ and $p(V_{j}^{\left(n\right)})$ are, respectively, the joint and marginal probability mass functions of vectors $V_{i}$ and $V_{j}$ ($V_{i}^{\left(m\right)}\in V_{i}$ and $V_{j}^{\left(n\right)}\in V_{j}$), and are defined as follows:

(45) $$p\left(V_{i}^{\left(m\right)}\right)=\frac{F_{a}\left(V_{i}^{\left(m\right)}\right)}{N}\;\mathrm{and}\;p\left(V_{i}^{\left(m\right)},V_{j}^{\left(n\right)}\right)=\frac{F_{a}\left(V_{i}^{\left(m\right)},V_{j}^{\left(n\right)}\right)}{N},$$

where $F_{a}$ represents the frequency of occurrence. Note that in practice, to calculate quantity $I(V_{i};V_{j})$, we adopt an approach based on a three-dimensional (3D) histogram of the data [49]. This approach involves discretizing the data space into three-dimensional bins, where each dimension corresponds to two variables, namely melanin associated with either oxyhemoglobin or deoxyhemoglobin. Subsequently, we count the number of occurrences of each combination of values in each of these bins, giving rise to a 3D histogram. From this histogram, we can extract the joint and marginal probabilities needed to calculate the mutual information. Finally, in the case of artificial dermatological images, as in [47], we also use a numerical performance measurement criterion based on the comparison of the estimated sources with the true sources, which are known in advance. The test results obtained for the two cases of dermatological images (real and artificial) are presented, respectively, in Section 3.1 and Section 3.2.

### 3.1. Real Dermatological Images

**Figure 3:**
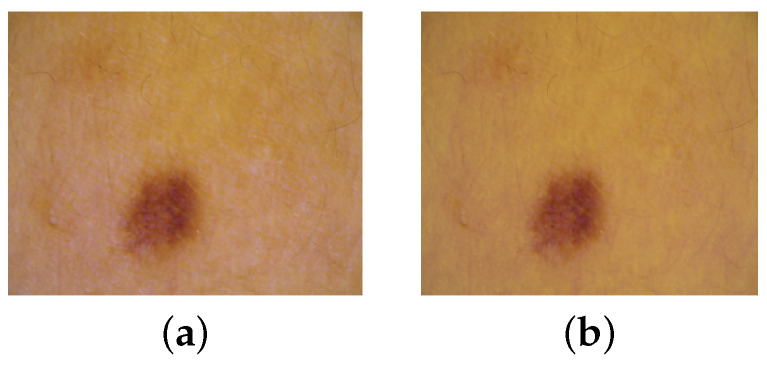
Processed dermatological image: (**a**) Image with specular reflection, (**b**) Image without specular reflection.

In this section, we examine the performances of our two new methods, *BCSnmf-Irgb* and *BCS-Irgb*, and that of method *BCS-rgb* using a database of real dermatological images. Developed by O. Lézoray et al. [50] and accessible via [51], this database consists of 30 multispectral dermatological images of patients with melanoma skin disease. The images were acquired as part of the Melascan project through collaboration with three companies—Intuiskin, Newton and Cynove—that specialize in developing imaging devices for skin analysis. More specifically, Intuiskin, based in Grenoble, designed a camera capable of capturing images under two different lighting conditions: white light and infrared. When the dermatologist activates the device, two consecutive images are recorded, each under one of the lighting modes. Intuiskin provided O. Lézoray et al. [50] with images of 30 skin lesions, captured in both visible light and infrared, with a resolution of 800×600 pixels. These multispectral dermatological images, accessible via [51], serve as the basis for our tests. To enhance the quality of the images, we first addressed the issue of specular reflection. We eliminated these reflections by detecting the affected areas using the method proposed in [13] and filling these areas with neighboring pixels through an interpolation technique (see Figure 3). Then, we applied these three methods to the entire set of 30 available images. For each image, we evaluated the performances qualitatively by referring to the visual analysis of the estimated chromophore concentrations and quantitatively by calculating the mutual information between these concentrations.

#### 3.1.1. Qualitative Study of Performances

**Figure 4:**
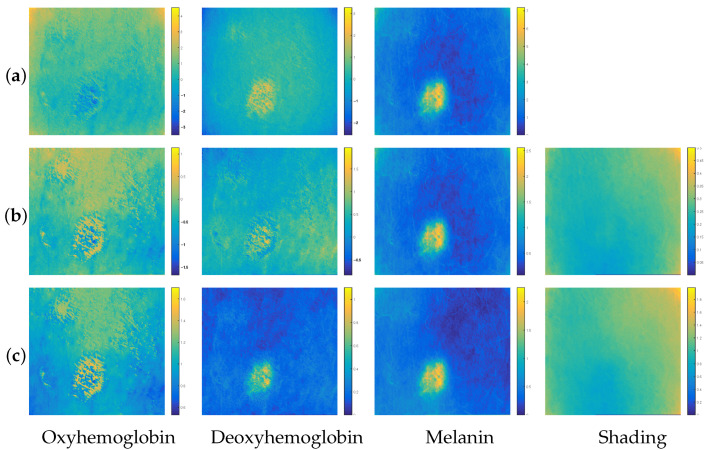
Contributions of chromophores and shading estimated by each of the three methods: (**a**) *BCS-rgb*, (**b**) *BCS-Irgb* and (**c**) *BCSnmf-Irgb*.

The tests we carried out on the entire set of 30 available images showed that our two methods, *BCSnmf-Irgb* and *BCS-Irgb*, are more performant than method *BCS-rgb*. Indeed, by referring to the visual analysis of the estimated concentrations by each of the three methods, we observed that the concentrations of the three chromophores estimated by method *BCS-rgb* are always affected by the concentration of shading. To illustrate this observation, and knowing that the results obtained are more or less similar from one image to another, we represent in Figure 3 one of the 30 images processed, and in Figure 4 the contributions of the chromophores and shading estimated by each of the three methods for this image.

From Figure 4, we can see that our methods, *BCSnmf-Irgb* and *BCS-Irgb*, are more performing than method *BCS-rgb*. Indeed, on the one hand, knowing that the presence of shading in a dermatological image is generally more important at its edges (we recall that the presence of shading in an image of a skin area is linked to the geometry and the variation of light incident on the surface of the skin), we deduce that the contributions of shading estimated by methods *BCS-Irgb* and *BCSnmf-Irgb* are more credible. We can see then that the concentration of oxyhemoglobin obtained by method *BCS-rgb* was poorly estimated because it is affected by the shading which inevitably alters the expected distribution for this chromophore in the case of melanoma and subsequently biases the identification of this disease.

On the other hand, we observe that the concentrations of oxyhemoglobin and deoxyhemoglobin estimated by method *BCS-Irgb* present negative values, which is not physically acceptable since a concentration is always positive or zero. This is not the case for the four concentrations (including those of the three chromophores) provided by our new method, *BCSnmf-Irgb*, which are always positive due to the fourth step of NMF, and thus the most credible. This demonstrates the relevance of this fourth step of our new global method, *BCSnmf-Irgb*, compared to our partial method, *BCS-Irgb*, that we proposed in [36].

#### 3.1.2. Quantitative Study of Performances

In order to evaluate the performance obtained numerically, for all the images, we refer to the calculation of mutual information to measure the independence between the different concentrations estimated for the chromophores. Indeed, we recall that the hypothesis of independence between chromophores is exploited both by our method and by all existing Blind Chromophore Separation (BCS) methods [22,23,24,26]. In other words, the aim is to measure the independence between the estimated chromophores with or without taking shading into account. To achieve this, we compare the mutual information between the chromophores estimated by method *BCS-rgb* (which neglects shading) with that between the chromophores estimated by our method at the stage of its third step, i.e., by our method *BCS-Irgb*. We specify that it is not relevant to be interested in the estimation of the mutual information between the chromophores after the fourth step of NMF in our method since, as mentioned in Section 2.4, the latter provides them with an update that could decrease their degree of independence achieved after Step 3.

Thus, for each of these two methods *BCS-Irgb* and *BCS-rgb*, we estimate the mutual information between the concentrations of oxyhemoglobin and melanin ($Y_{1}\left(u\right)$ and $Y_{3}\left(u\right)$) and between those of deoxyhemoglobin and melanin ($Y_{2}\left(u\right)$ and $Y_{3}\left(u\right)$) denoted, respectively, as $I(Y_{1}\left(u\right);Y_{3}\left(u\right))$ and $I(Y_{2}\left(u\right);Y_{3}\left(u\right))$, which are defined by Equation (44). We then define their mean denoted as $I_{m}$ as follows:

(46) $$I_{m}=\frac{1}{2}\left\{I\left(Y_{1}\left(u\right);Y_{3}\left(u\right)\right)+I\left(Y_{2}\left(u\right);Y_{3}\left(u\right)\right)\right\}.$$

Furthermore, in order to have an idea of the degree of improvement in independence regarding the four base mixtures $X_{i}\left(u\right)$ (provided by the four IRGB spectral bands), we also provide an estimation of the quantity $I_{m}$ at the input of our system (i.e., before separation), which involves all quantities $I(X_{i}\left(u\right);X_{j}\left(u\right))$ for i<j,(i,j=1,2,3,4). We then calculate the mean value as well as the standard deviation of the mutual information $I_{m}$, denoted, respectively, as ${\bar{I}}_{m}$ and σ, over the set of 30 processed images before and after separation by each of the two methods. The obtained results are presented in Table 1.

**Figure 5:**
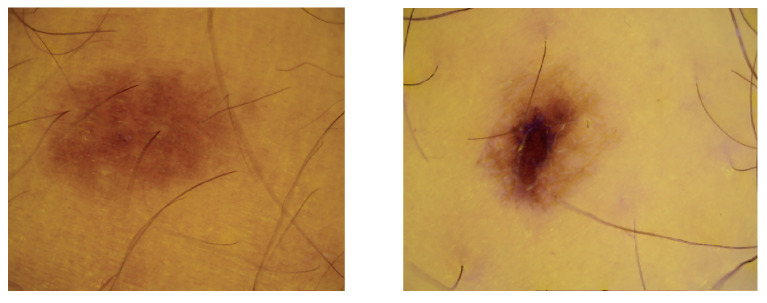
Example of dermatological images containing hair.

From Table 1, we observe that our method *BCS-Irgb* provides a mutual information value (equal to 0.14) much lower than that of method *BCS-rgb* (equal to 0.22) and that obtained at the input between the different spectral bands IRGB (equal to 0.26). Since the mutual information is inversely proportional to the independence, we deduce that our method *BCS-Irgb* provides chromophore concentrations that are much more independent than those provided by method *BCS-rgb*. This result demonstrates the relevance of our key idea of considering shading as a fully fledged source and then exploiting the four spectral bands IRGB instead of the three classical RGB spectral bands. Indeed, the fact that method *BCS-rgb* does not improve the degree of independence (compared to that obtained at the input between the four spectral bands IRGB) is simply explained by the fact that the estimated concentrations of the different chromophores are generally affected by shading, which has not been taken into account in the mixing model at the beginning. In other words, in the estimated concentration of each chromophore, there is a more or less significant residue of shading. However, it should be noted that the non-zero standard deviation we obtained for our method *BCS-Irgb* reflects the existence of some images for which the mutual information $I_{m}$ is relatively higher than 0.14, which means that the estimated chromophore concentrations corresponding to these images are relatively less independent. We indeed observed that these particular images all contain a non-negligible level of hair. This result, as expected, is explained by the fact that these images do not perfectly satisfy our initial mixing model. An example of these images is provided in Figure 5.

On the other hand, in order to quantify the contribution of the fourth step of NMF of our global method *BCSnmf-Irgb* in terms of performance, compared to our partial method *BCS-Irgb* [36], we propose this time to refer to the estimated mixing matrix M. We recall that the coefficients $m_{ij}$ of this matrix are closely related to the absorption coefficients of the chromophores (the coefficients $m_{ij}$ are in fact proportional to the absorption coefficients of the chromophores, as mentioned in Section 2.3) which are always positive and depend on the wavelength as shown in Figure 1. Thus, by exploiting on the one hand the hypothesis that the absorption coefficients of oxyhemoglobin and deoxyhemoglobin in the two red and infrared bands are almost zero (according to this Figure 1), and on the other hand the hypothesis that the contribution of shading varies very little from one spectral band to another [37], we can very well take as a criterion for measuring the performances degree of coherence of the numerical values obtained for the coefficients $m_{ij}$ with these working hypotheses. To achieve this, given that we cannot present here all the mixing matrices corresponding to the 30 images tested, we provide in Table 2 the matrix M obtained for the image used in Figure 3 (referred to as Image (a)), and on the other hand the mean matrix, denoted as $\bar{M}$, of all the estimated matrices (corresponding to the 30 images tested).

From the estimations of the two matrices M and $\bar{M}$ provided in Table 2, we can see that the fourth step of *NMF* of our global method *BCSnmf-Irgb* is of great utility. Indeed, we can see that some coefficients of the matrix M estimated by method *BCS-Irgb* are negative, which is intolerable, since they are directly related to the absorption coefficients of different chromophores that are always positive or zero. This result, which is due to errors in estimating the coefficients of the matrix M, is expected since the independence and sparsity hypotheses on which the method *BCS-Irgb* is based are not perfectly verified (as mentioned in Section 2.4). In contrast, the coefficients of the matrix M estimated using our global method *BCSnmf-Irgb* are all positive and are much more in line with the working hypotheses. Indeed, on the one hand, the coefficients $m_{ij}\;\left(i=3,4\;\mathrm{and}\;j=1,2\right)$ associated with oxyhemoglobin and deoxyhemoglobin, respectively, are almost zero, which is perfectly consistent with the information provided by the experimental curves in Figure 1. On the other hand, the coefficients of the fourth column associated with shading are slightly different, which is consistent with the fact that physically the contribution of shading varies very little from one spectral band to another. Furthermore, all these observations are more or less valid for all the images tested, according to the estimation of the matrix $\bar{M}$ provided in Table 2. Indeed, despite the existence of some images which present particularities related to the existence of hairs (such as the one presented in Figure 5), the difference between the values obtained for the $m_{ij}$ coefficients and the expected values (similar to those of Image (a)) remains very reasonable. We therefore conclude that our global method *BCSnmf-Irgb* is quite robust regarding this type of particularity.

### 3.2. Artificial Dermatological Images

We first recall that in the source separation community, it is usual to study the performance of any BSS method on artificial mixtures of artificial sources that are known in advance. This allows to compare the sources estimated by any BSS method to the true sources (which are known) by using a numerical criterion based on a measure of the deviation between the two, and thus to be able to precisely quantify the separation quality of this method. Furthermore, this also enables us to ensure that if the working hypotheses are verified by the sources involved, then their separation by the proposed method is guaranteed. In this regard, it is relevant to validate our new BCS method (with our two methods *BCS-Irgb* and *BCSnmf-Irgb*) also on artificial dermatological images. However, since there is no database of this type in the literature, we proposed in this article to artificially simulate our own dermatological images by adopting a well-studied protocol to generate the four sources $S_{j}\left(u\right)$ which are nothing other than the concentrations of the three chromophores and shading. Indeed, based on the principle that the simulated dermatological image should be as close as possible to a real dermatological image, we exploited physiological knowledge about human skin with its various diseases as well as about chromophores [52] while respecting, as much as possible, the hypotheses made about their concentrations in terms of positivity, independence and sparsity. However, we note that we deliberately avoided using chromophore concentrations estimated from real images, on the one hand to avoid simulating images that are identical to real images that have already been processed, and on the other hand to be able to better control chromophores in terms of working hypotheses. In this regard, after testing a multitude of configurations for simulating the four concentrations, which give rise to the four spectral bands *IRGB* of the simulated image through the mixture model (6), we selected the following configuration:

- Take for the concentration of melanin ($S_{3}\left(u\right)$) a perfectly realistic version corresponding to the concentration estimated from one of the real melanoma dermatological images processed in Section 3.1. Indeed, we observed that this choice allowed us to obtain artificial images that are quite close to real ones. This is because melanin is the most visible chromophore to the eye compared to the other two chromophores, as it is present in the first layer of the skin. Knowing that the concentrations of the other two chromophores as well as the shading were generated artificially as explained below.
- Simulate artificially a concentration of oxyhemoglobin ($S_{1}\left(u\right)$) while respecting the working hypotheses, i.e., the hypothesis of its sparsity according to Equation (24) and the hypothesis of its independence from melanin ($S_{3}\left(u\right)$) according to Equation (18).
- Simulate artificially a concentration of deoxyhemoglobin ($S_{2}\left(u\right)$) by exploiting the simulated concentration of oxyhemoglobin ($S_{1}\left(u\right)$). We based this on the fact that the concentrations of these two chromophores combine to produce the total concentration of hemoglobin [37], which we set to one in all pixels of the image for simplicity. This allowed us to simultaneously satisfy our two working hypotheses, i.e., the hypothesis of complementary sparsity of these two chromophores $S_{1}\left(u\right)$ and $S_{2}\left(u\right)$, ruled by two Equations (24) and (25), and the hypothesis of their independence from melanin ($S_{3}\left(u\right)$) ruled by Equation (18).
- Simulate artificially a shading concentration ($S_{4}\left(u\right)$) that is as independent as possible of the three chromophores, as assumed in the working Hypotheses (11) and (18). Knowing that shading, which represents the variation in the flux of incident light on the surface of the skin and also depends on the geometry of that surface, is always much greater at the edges of the dermatological image, the values of its concentration $S_{4}\left(u\right)$ that we simulated are high at the edges of the image and progressively decrease as we move toward the center of the image. However, we note that we encountered difficulty in perfectly satisfying all the working hypotheses simultaneously to simulate our four concentrations. We observed that increasing the degree of independence between our simulated concentrations comes at the expense of the sparsity of $S_{1}\left(u\right)$ and $S_{2}\left(u\right)$.

To artificially simulate a mixing matrix M, we exploited, on the one hand, the absorption spectra of the three chromophores provided in Figure 1, and on the other hand the expressions of some mixing matrices that we estimated in the case of real dermatological images. In this regard, here is the expression of the mixing matrix that served as a basis for generating other mixing matrices:

(47) $$M=\left(\begin{array}{cccc} 0.86 & \;0.70 & \;0.37 & \;0.25\\ 0.13 & \;0.29 & \;0.36 & \;0.26\\ 0 & \;0 & \;0.18 & \;0.23\\ 0 & \;0 & \;0.07 & \;0.24 \end{array}\right).$$

By exploiting the four simulated sources and the mixing matrix M, we simulate an artificial dermatological image using Equation (1), with G(λ)=2 and $E_{d}\left(\lambda \right)=1$ which represent the image acquisition conditions in our simulation.

**Figure 6:**
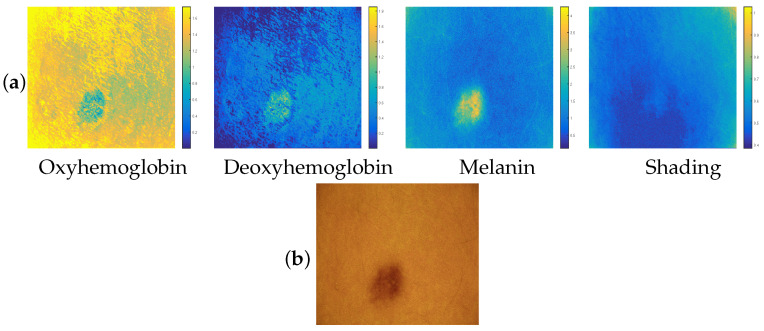
(**a**) Simulated contributions of the three chromophores and shading, (**b**) Resulting RGB dermatological image.

In order to have a testing protocol that is similar to that of real dermatological images, we simulated 30 artificial dermatological images. To generate a new artificial image, we varied the base concentration values as well as the coefficients of the base mixing matrix M by adding small randomly generated positive values to them (we added positive random values generated by MATLAB Online [53] using the rand command). In Figure 6, we provide one of our 30 artificial dermatological images, which we represent in the three visible RGB bands along with the contributions of three chromophores and shading that allowed us to generate it.

#### 3.2.1. Qualitative Study of Performances

**Figure 7:**
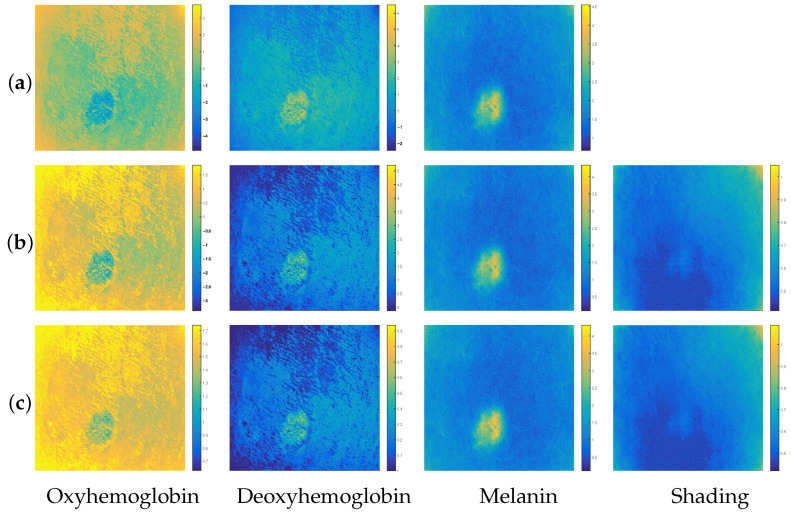
Contributions of the chromophores and shading estimated by each of the three methods: (**a**) *BCS-rgb*, (**b**) *BCS-Irgb* and (**c**) *BCSnmf-Irgb*.

After processing all 30 simulated images, we observed that the results obtained are roughly similar from one simulated image to another, based on the visual analysis of the concentrations estimated by each of the three methods *BCS-rgb*, *BCS-Irgb* and *BCSnmf-Irgb*. To illustrate this observation, Figure 7 shows the contributions of the chromophores and shading estimated by each of these three methods.

From Figure 7, we can see that our methods *BCS-Irgb* and *BCSnmf-Irgb* perform better than the method *BCS-rgb*. Indeed, referring to the visual analysis of the contributions estimated by each of these methods, we see that the contributions estimated by method *BCS-rgb* are the furthest from the simulated ones (which are now known and shown in Figure 6). This is particularly noticeable in the contribution of deoxyhemoglobin. All these observations are confirmed by referring to the color bars that provide numerical information on the concentration range of each chromophore. Moreover, these color bars also allow us to conclude that method *BCSnmf-Irgb* performs better than method *BCS-Irgb*, which provides an estimation of the oxyhemoglobin contribution indicating the presence of negative values in the estimated concentration of this chromophore. Therefore, method *BCSnmf-Irgb* provides the best estimations of the chromophore contributions compared to the simulated contributions, which is consistent with the results obtained in the case of real images.

#### 3.2.2. Quantitative Study of Performances

For the quantitative performance study, we begin by numerically evaluating the performance of methods *BCS-rgb* and *BCS-Irgb* exactly as we did for the real images, i.e., by referring to the calculation of the mutual information. We then calculate the mean and standard deviation of the mutual information $I_{m}$ (defined by Equation (46)) over all 30 simulated images, both at the input (between the four spectral bands Irgb) and at the output between the different contributions estimated by each of the two methods. Since we now know the simulated contributions, we additionally calculate the mutual information between them using Equation (46) for $Y_{j}\left(u\right)=S_{j}\left(u\right)$. The results obtained are presented in Table 3.

From Table 3, we observe that our method *BCS-Irgb* provides a mean value and a standard deviation of mutual information (equal to 0.17 and $1.1\times 10^{-3}$, respectively) that are lower than those provided by method *BCS-rgb* (equal to 0.20 and 0.01, respectively). Since our two values of $I_{m}$ and σ are closest to those obtained at the input between the simulated contributions (equal to 0.15 and $0.68\times 10^{-3}$, respectively), we deduce that our method *BCS-Irgb* performs better than method *BCS-rgb* in terms of independence between the estimated concentrations of the chromophores.

On the other hand, as mentioned at the beginning of Section 3.2, it is usual to numerically evaluate the performance of any BSS method by comparing the estimated sources to the simulated ones using a criterion that measures the deviation between them. Therefore, we propose to compare each simulated source $S_{j}\left(u\right)$ with its estimated $Y_{j}\left(u\right)$ by each of the three methods *BCS-rgb*, *BCS-Irgb* and *BCSnmf-Irgb* using the most popular criterion in the BSS community [17], namely the Signal to Interference Ratio (SIR), denoted $SIR_{j}$ and defined by the following equation:

(48) $$SIR_{j}=10\cdot log_{10}\left(\frac{E\left[S_{j}{\left(u\right)}^{2}\right]}{E\left[{\left(S_{j}\left(u\right)-Y_{j}\left(u\right)\right)}^{2}\right]}\right),\;j\in \left[1,4\right],$$

where $Y_{j}\left(u\right)$ represents the estimation of the source $S_{j}\left(u\right)$, knowing that both $Y_{j}\left(u\right)$ and $S_{j}\left(u\right)$ are previously centered and normalized to have the same variance. We then calculate the mean and standard deviation of $SIR_{j}$ denoted, respectively, ${\bar{SIR}}_{j}$ and $\sigma_{j}$, over the 30 available realizations. The results obtained for the three methods *BCS-rgb*, *BCS-Irgb* and *BCSnmf-Irgb* are presented in Table 4.

From Table 4, we observe that our two methods *BCS-Irgb* and *BCSnmf-Irgb* perform better than method *BCS-rgb* in terms of SIR. Indeed, we can see that our partial method *BCS-Irgb* surpasses method *BCS-rgb* as it provides $SIR_{j}$ values that are significantly higher than those provided by the latter, with much lower standard deviations, for all three chromophores. However, it should be noted that concentrations $S_{1}\left(u\right)$ and $S_{2}\left(u\right)$ (of oxyhemoglobin and deoxyhemoglobin) are estimated with significantly lower performance compared to concentrations $S_{3}\left(u\right)$ and $S_{4}\left(u\right)$ (of melanin and shading) by our method *BCS-Irgb*. From this result, we can deduce that the hypothesis of sparsity at the level of $S_{1}\left(u\right)$ and $S_{2}\left(u\right)$ as well as the hypothesis of their independence regarding $S_{3}\left(u\right)$ and $S_{4}\left(u\right)$ required by this method are not verified to the same degree as the hypothesis of independence between $S_{3}\left(u\right)$ and $S_{4}\left(u\right)$ at the level of the artificial dermatological images that we simulated. Nevertheless, we observed an improvement in the estimation of these two concentrations ($S_{1}\left(u\right)$ and $S_{2}\left(u\right)$) by our global method *BCSnmf-Irgb*, confirming the contribution of the NMF step, which constitutes the fourth step of this method.

## 4. Discussion and Conclusions

In this article, we proposed a new method for the Blind Separation of principal Chromophores (BCS) of the skin (i.e., melanin, oxyhemoglobin and deoxyhemoglobin) from multispectral dermatological images. The novelty of our method lies, on the one hand, by taking into account the shading during modelling as a fully fledged source in the same way as the three chromophores (unlike all existing methods that neglect it or consider that its contribution to the different spectral bands of the image is exactly the same), and on the other hand in the approach we adopted to separate them. We modeled the image provided by each spectral band as a mixture of four sources corresponding to the three main chromophores in addition to the shading. Thus, in addition to the three classic visible RGB spectral bands, we used a fourth spectral band to have as many mixtures as the unknown sources to identify. Hence the necessity of having at least four bands of multispectral images for our new method, which proceeds in four steps. Our first step is based on the key idea of exploiting the Infrared band as the fourth spectral band, and the fact that at this band as well as the Red band, only melanin and shading are present, allowing us to separate them by using only these two bands. In our second step, these two sources are subtracted from the mixtures provided by the green and blue bands to obtain two new mixtures that contain only oxyhemoglobin and deoxyhemoglobin. Assuming that these latter sources are sparse and independent of melanin and shading, we proceed to their separation in the third step of our method. Based on NMF, the fourth step reduces the errors in the source estimates obtained in the first three steps, which are due to the fact that the working hypotheses are never perfectly verified in practice. To demonstrate the interest of this fourth NMF step, we proposed two versions of our new BCS method, which exploits the four IRGB spectral bands: a first version with only three steps, which we named *BCS-Irgb* and which can qualify as a partial method, then a second version including the NMF step which we named *BCSnmf-Irgb*, and which we can qualify as a global method. On the other hand, as our aim is to demonstrate the relevance of taking shading into account, we compared our performances with those obtained by neglecting it, which corresponds to the use of the method we named *BCS-rgb*, which consists of separating the three chromophores by exploiting only the three classic RGB spectral bands (which is adopted by almost all existing methods). Knowing that we have studied the performances qualitatively and quantitatively on both real and artificial multispectral dermatological images, we found that

- **For real dermatological images**: The qualitative performance study based on visual analysis of the estimated concentrations of the three chromophores showed that our two methods *BCS-Irgb* and *BCSnmf-Irgb* perform better than method *BCS-rgb* since the latter produced concentrations affected by shading. Furthermore, this qualitative study clearly demonstrated that the fourth NMF step in our method is of great importance, as it significantly improved the concentrations estimated by method *BCS-Irgb*. The quantitative performance study, based on measuring the independence between the estimated concentrations of the chromophores (using mutual information as the numerical criterion), confirmed the results of the qualitative study, showing that the concentrations estimated by our method *BCS-Irgb* are significantly more independent than those provided by method *BCS-rgb*.
- **For artificial dermatological images:** The results obtained are generally in perfect coherence with those obtained for real dermatological images, both qualitatively and quantitatively. Furthermore, since in this case, we know in advance the concentrations that produced these artificial dermatological images, we were able to evaluate the performances even more precisely by comparing these known concentrations with those estimated by the three tested methods (using SIR as the numerical criterion). This comparison once again confirmed the superiority of our two methods over method *BCS-rgb*.

We are, however, aware that some aspects of our work require further study and thus open up various perspectives for us. Indeed,

- The results we presented in this paper correspond to 30 real multispectral dermatological images of melanoma [50] and 30 artificial multispectral dermatological images of melanoma. It is therefore desirable to validate these results on more databases of real dermatological images, potentially covering different skin diseases, and on a much larger database of artificial dermatological images. Nevertheless, based on our tests, we point out that simulating multispectral dermatological images that are as realistic as possible is not an easy task and thus deserves much more in-depth study. We believe that a database of such artificial images will be of great use to researchers in the field of dermatology.
- Knowing that the concentrations of the three main chromophores estimated separately from dermatological images have been used for the identification of some skin diseases [5,6,36], and given that our study in this article has shown that taking shading into account in our mixing model significantly improves the quality of these concentrations, it is desirable to validate the contribution of this improvement in terms of skin disease identification.
- We observed that the presence of disruptive elements such as hair in a dermatological image degrades our performance. Therefore, the use of existing preprocessing methods or the development of new methods capable of reducing the artifacts caused by these various disruptive elements is desirable.

## Figures and Tables

**Table 1:** Mean value and standard deviation of the mutual information $I_{m}$ at the input (between the 4 *Irgb-bands*), then at the output using methods *BCS-rgb* and *BCS-Irgb*.

	Input	Output	
Method	*Irgb-bands*	*BCS-rgb*	*BCS-Irgb*
${\bar{I}}_{m}$	0.26	0.22	**0.14**
σ	0.02	0.05	**0.04**

**Table 2:** Estimation of the mixing matrix M for image (a) (first row) and the mean matrix $\bar{M}$ over all images (second row) using our two methods *BCS-Irgb* and *BCSnmf-Irgb*.

Method	*BCS-Irgb*	*BCSnmf-Irgb*
M for image (a)	$\left(\begin{array}{cccc} \;1.00 & \;1.00 & \;0.08 & \;0.001\\ \;0.38 & \;0.75 & \;0.12 & -0.02\\ -0.00 & -0.00 & \;0.06 & -0.002\\ -0.00 & -0.00 & -0.00 & \;0.09 \end{array}\right)$	$\left(\begin{array}{cccc} 0.82 & \;0.28 & \;0.30 & \;0.29\\ 0.17 & \;0.71 & \;0.35 & \;0.20\\ 0 & \;0 & \;0.29 & \;0.21\\ 0 & \;0 & \;0.03 & \;0.27 \end{array}\right)$
$\bar{M}$ for all images	$\left(\begin{array}{cccc} \;1.00 & \;1.00 & \;0.18 & \;-0.003\\ \;0.10 & \;0.30 & \;0.32 & \;-0.03\\ -0.00 & \;-0.00 & \;0.19 & \;0.001\\ -0.00 & \;-0.00 & \;0.03 & \;0.04 \end{array}\right)$	$\left(\begin{array}{cccc} 0.84 & \;0.26 & \;0.26 & \;0.20\\ 0.14 & \;0.72 & \;0.33 & \;0.24\\ 0 & \;0 & \;0.36 & \;0.25\\ 0 & \;0 & \;0.02 & \;0.30 \end{array}\right)$

**Table 3:** Mean and standard deviation of mutual information $I_{m}$ at the input (between simulated contributions and between spectral bands) and at the output between chromophores estimated by each of the methods *BCS-rgb* and *BCS-Irgb*.

	Simulated Contributions	Mixtures *Irgb-Bands*	Estimated Contributions	
			Method *BCS-Rgb*	Method *BCS-Irgb*
${\bar{I}}_{m}$	0.15	1.96	0.20	**0.17**
σ	0.68 $\times 10^{-3}$	0.15	0.01	$1.1\times 10^{-3}$

**Table 4:** Mean and standard deviation of $SIR_{j}$ in dB using methods *BCS-rgb*, *BCS-Irgb* and *BCSnmf-Irgb*.

	${\bar{\mathrm{SIR}}}_{j}$ (dB)				$\sigma_{j}$ (dB)			
Method	$S_{1}\left(u\right)$	$S_{2}\left(u\right)$	$S_{3}\left(u\right)$	$S_{4}\left(u\right)$	$S_{1}\left(u\right)$	$S_{2}\left(u\right)$	$S_{3}\left(u\right)$	$S_{4}\left(u\right)$
*BCS-rgb*	13.42	13.61	15.51	–	4.52	4.75	1.49	–
*BCS-Irgb*	26.01	24.47	35.15	40.12	0.12	0.31	0.63	1.29
*BCSnmf-Irgb*	**33.14**	**26.00**	**35.15**	**40.12**	3.00	0.81	0.63	1.29

## Data Availability

The datasets generated during the current study are available in [50] and the code is available from the corresponding author upon reasonable request.
